# Defining an Essence of Structure Determining Residue Contacts in Proteins

**DOI:** 10.1371/journal.pcbi.1000584

**Published:** 2009-12-04

**Authors:** R. Sathyapriya, Jose M. Duarte, Henning Stehr, Ioannis Filippis, Michael Lappe

**Affiliations:** Structural Genomics/Bioinformatics Group, Otto Warburg Laboratory, Max Planck Institute for Molecular Genetics, Berlin, Germany; National Cancer Institute, United States of America and Tel Aviv University, Israel

## Abstract

The network of native non-covalent residue contacts determines the three-dimensional structure of a protein. However, not all contacts are of equal structural significance, and little knowledge exists about a minimal, yet sufficient, subset required to define the global features of a protein. Characterisation of this “structural essence” has remained elusive so far: no algorithmic strategy has been devised to-date that could outperform a random selection in terms of 3D reconstruction accuracy (measured as the Ca RMSD). It is not only of theoretical interest (i.e., for design of advanced statistical potentials) to identify the number and nature of essential native contacts—such a subset of spatial constraints is very useful in a number of novel experimental methods (like EPR) which rely heavily on constraint-based protein modelling. To derive accurate three-dimensional models from distance constraints, we implemented a reconstruction pipeline using distance geometry. We selected a test-set of 12 protein structures from the four major SCOP fold classes and performed our reconstruction analysis. As a reference set, series of random subsets (ranging from 10% to 90% of native contacts) are generated for each protein, and the reconstruction accuracy is computed for each subset. We have developed a rational strategy, termed “cone-peeling” that combines sequence features and network descriptors to select minimal subsets that outperform the reference sets. We present, for the first time, a rational strategy to derive a structural essence of residue contacts and provide an estimate of the size of this minimal subset. Our algorithm computes sparse subsets capable of determining the tertiary structure at approximately 4.8 Å Ca RMSD with as little as 8% of the native contacts (Ca-Ca and Cb-Cb). At the same time, a randomly chosen subset of native contacts needs about twice as many contacts to reach the same level of accuracy. This “structural essence” opens new avenues in the fields of structure prediction, empirical potentials and docking.

## Author Summary

A protein structure can be visualized as a network of non-covalent contacts existing between amino acids. But not all such contacts are important structural determinants of a protein. We have attempted to identify a subset of amino acid contacts that are essential for reconstructing protein structures. Initially, we followed random sampling of contacts and tested their efficacy to successfully represent the three-dimensional structure. Further, we also developed an algorithm that selects a subset of amino acid contacts from proteins based on the sequence and network properties. The subsets picked by our algorithm represent protein three-dimensional structure better than random subsets, thereby offering direct evidence for the existence of a structural essence in protein structures. The identification of such structure-defining subsets finds application in experimental and computational protein structure determination.

## Introduction

**Figure 1:**
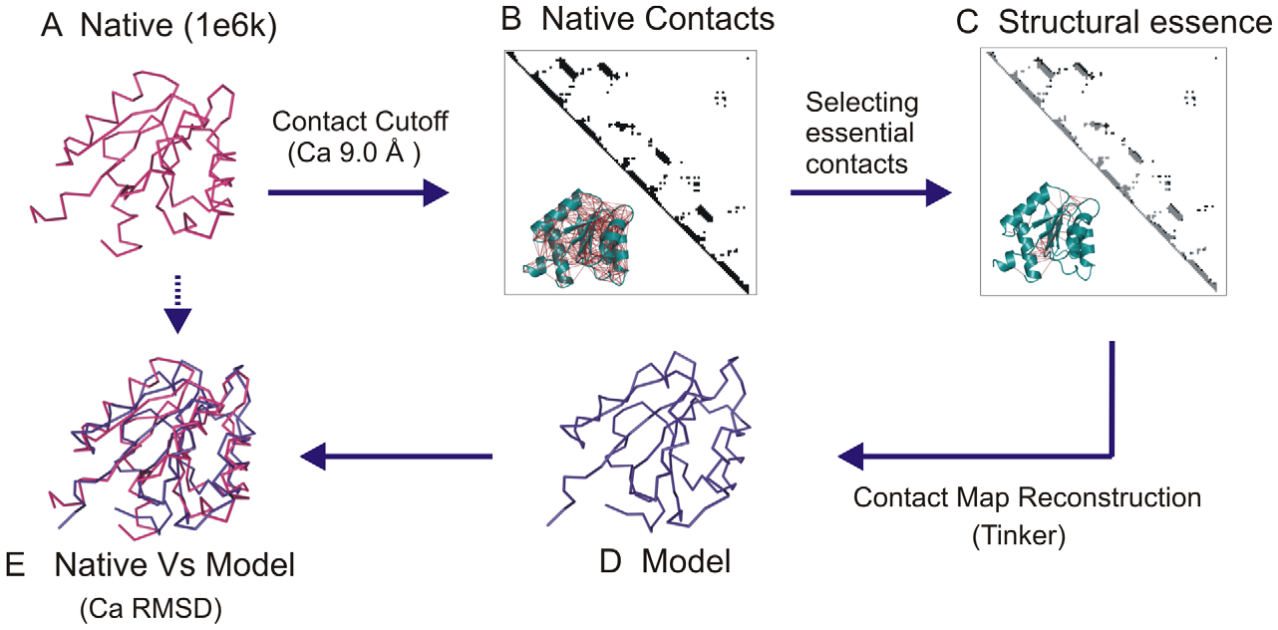
The concept of structural essence. The concept of a minimal set of contacts essential for the reconstruction of the three-dimensional structure is elucidated with an example of CheY (1e6k). A The native structure of 1e6k is shown in ribbon representation (pink). B The Ca contacts are visualized in a contact map. The inset highlights all the Ca contacts (red) on the cartoon representation. C A subset selected from the native contact map is highlighted (black). The inset shows the selected subset mapped onto the structure. D The structure reconstructed from the selected subset is shown in ribbon representation (blue). E The superposition of the native and the reconstructed structures. The reconstruction accuracy is measured as the Ca RMSD of the superposition of the native structure and the reconstructed model.

The native structure of a protein is held intact by the complex and cooperative interplay of residue interactions. While a network of amino acid contacts is well defined given a native structure, it remains an open question if all the contacts are equivalent in terms of their contribution to the structural integrity. In part, this question has been addressed by studies where a partial contact network of a native structure is embedded into the three-dimensional space [1],[2]. The resultant root mean square deviation (RMSD) of the embedding from the native structure quantifies the information content of the selected subset (Figure 1).

Such a line of investigation also represents a logical extension to the current trends in structural biology. While most of the three-dimensional structures of proteins in the PDB are identified by X-ray crystallography and NMR, new experimental methods like EPR aim to broaden the horizon of structural proteomics and cover the protein universe [3]–[5]. In spirit, these techniques are similar to established NMR spectroscopic methods as they yield information about inter-residue proximity constraints. From a sufficient number of such experimentally derived constraints, the tertiary structure of the protein can be identified [6]. Identifying a minimal set of structure determining distance constraints a-priori from the sequence would not only minimize the experimental efforts, but would in fact imply a solution to the protein folding problem. As an intermediate step in this direction, analysing minimal subsets of structure determining contacts in known structures promises to provide preliminary insights into the question what the features of such an essential subset of contacts might be.

For several years, the distance constraints and other stereo chemical and biophysical restraints have been employed in computational restraint-based protein modelling [7]–[13]. Specifically, a selected subset of native contacts is considered as distance constraints that efficiently define the fold and reconstruct the tertiary structure of the protein [14]. In order to obtain coordinates consistent with a given set of distance constraints, we implemented a contact map reconstruction method based on distance geometry [15]. Using the complete set of native contacts of a known protein structure as input, the reconstruction provides models that are within 2.0 Å Ca RMSD from the native structure (Jose M Duarte et al. unpublished data). The other existing implementations of 3D reconstruction from contact maps are based on methods such as Discrete Molecular Dynamics (DMD) [16], singular value decomposition [2],[17]. The reconstruction accuracies of the alternate reconstruction methods vary and different contact definitions and datasets make a direct comparison difficult. Despite the quantitative differences in reconstruction accuracy, we reproduce qualitatively the same non-linear relationship of Ca RMSD with fraction of native contacts. In all such studies, below a certain fraction of the native contacts, the reconstruction accuracy deteriorates rapidly. With our reconstruction pipeline we could go as low as 20–30% of the native contacts (Ca-Ca, Cb-Cb) and still obtain an average reconstruction accuracy of ∼4 Å Ca RMSD. In summary, we confirmed that a contact map is highly redundant and a subset of native contacts is sufficient to determine the structure up to experimental accuracy.

Together with an accurate 3D reconstruction method and the knowledge that a complete contact map is not required for recognizing the protein fold, the central question is to predetermine the nature and the number of ‘minimal distance constraints’ required to efficiently identify the tertiary structure of the protein (Figure 1). The current paper focuses on the methods used to derive a minimal set of contacts, the necessary and sufficient determinants to reconstruct any given protein fold, namely the ‘structural essence of a protein’. An independent study by Chen et al showed that randomly picked subset of contacts could be used to successfully reconstruct the three-dimensional structure of the protein [16]. They further claimed that subsets selected with a rational strategy could only reconstruct as good as the random subsets and not better. Here, for the first time we demonstrate that a structural essence exists and provide a constructive algorithm for its calculation. We also characterize the structural essence from different folds. The results of this study facilitate the choice of contacts to obtain better models from experimental and computational restrain-based protein modelling.

## Results

### Less is more in 3D reconstruction of protein structures

**Figure 2:**
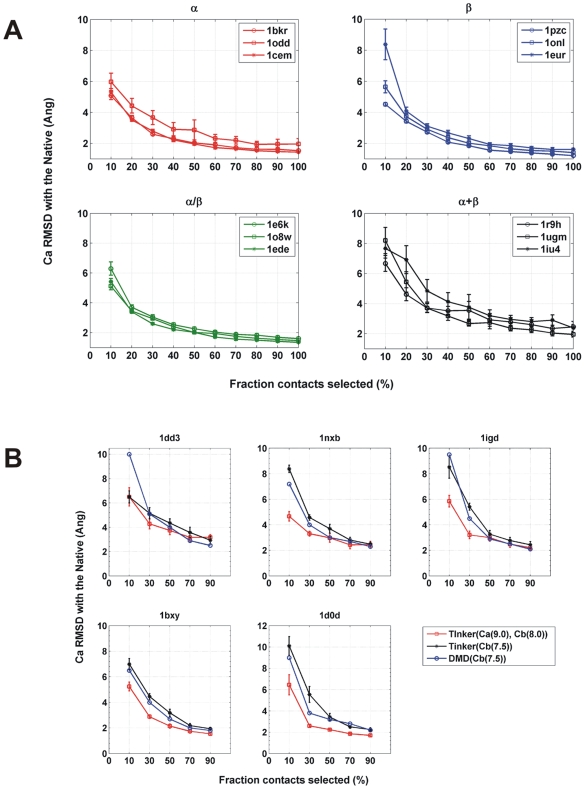
Subsets from random selection. A Increasing fractions of contacts (from 10% to 100%) are selected at random and reconstructed. Two independent random selections are performed for every fraction and the average Ca RMSD is reported for every protein in a SCOP class. Each class consists of three structures. In each class ‘*’ denotes proteins that are thrice as large as the other two proteins. B The reconstruction accuracies of the random subsets are compared between our method and Chen and co-workers. Five proteins (1dd3, 1nxb, 1igd, 1bxy, 1d0d) are selected from the Chen dataset and the random subsets are generated with (i) our contact definitions Ca 9.0 Å, Cb 8.0 Å (red) (ii) contact definition from Chen et al (Cb 7.5 Å) (black). Subsets from (i) and (ii) are reconstructed with Tinker (iii) The reconstruction accuracy from Chen et al (blue).

To verify that a subset of native contacts is sufficient to reconstruct the native structure, we chose increasing fractions (from 10%–90%) randomly from native contacts and measured their reconstruction accuracy (as Ca RMSD compared to the native structure). The reconstruction accuracies obtained are provided in Figure 2A. The dataset chosen for the study is given in Table 1. We find that in all the proteins from the dataset, the reconstruction with a fraction of native contacts yields structures close to the native structure. Specifically, the 30%–50% random subsets show reconstruction accuracies comparable to those obtained from the complete contact map. The negligible increase in Ca RMSD between the 20% and 30% subsets provides a direct estimate of the size of the minimal subset (Figure 2A).

**Table 1:** Dataset.

PDB	SCOP id	#Nodes	#Edges (Ca-Ca, Cb-Cb)	Size (% Ca-Ca, Cb-Cb contacts)	PI*
**All α**					
1bkr	a.40.1.1	109	553, 339	5.6, 9.3	2.14
1odd	a.4.6.1	118	508, 341	5.3, 8.8	1.84
1cem	a.102.1.2	363	2273, 1627	6.2, 11.6	1.53
**All β**					
1pzc	b.6.1.1	123	679, 481	5.4, 13.7	1.61
1onl	b.84.1.1	128	690, 510	5.7, 11.0	1.85
1eur	b.68.1.1	365	2244, 1567	6.7, 14.1	1.74
**α/β**					
1e6k	c.23.1.1	130	669, 503	4.3, 8.9	1.74
1o8w	c.47.1.10	146	722, 524	6.8, 12.8	3.13
1ede	c.69.1.8	310	1764, 1315	5.5, 11.8	1.69
**α + β**					
1r9h	d.26.1.1	135	629, 446	5.9, 9.9	1.26
1ugm	d.15.1.3	125	519, 379	5.6, 10.0	2.06
1iu4	d.3.1.8	331	1935, 1316	6.2, 11.6	1.51

*: PI for the cone-peeled subsets are calculated using Eq. (1). For the sake of comparison, the GDT_TS is given in for the cone-peeled and the random subsets.

The existence of the structural essence is observed as a common feature across different folds. However, the size of the subset varied with different SCOP classes as shown in Figure 2A. For proteins from the all α, all β and the α/β SCOP classes, as low as 20% of the native contacts are sufficient to obtain a structure within Ca RMSD of ≤4 Å to the native structure. However in the α+β class more contacts (30%) are required for acquiring the same reconstruction accuracy (Figure 2A) highlighting its higher topological complexity. Furthermore, it is worthwhile to note that 20% of contacts are sufficient for reconstruction across a range of protein sizes between 100 and 300 amino acids. Thus, there is a negligible effect of protein size on the reconstruction accuracy with our reconstruction method.

The performance comparison of our contact definition and our reconstruction pipeline with other existing methods of contact map reconstruction namely FT-COMAR [1], DMD [16] reveals differences in the reconstruction accuracies and the size of the minimal subsets. In order to systematically compare the differences, we have taken the dataset from Chen et al (henceforth called Chen-set) and repeated the reconstructions with our method and contact definition (for details see methods). The results are shown in the Figure 2B. We find that the overall profile of the reconstruction accuracy of different random subsets from the Chen-set did not vary considerably between our method and DMD. However, there are differences observed in the size of the minimal subset. In contrast to the 70% contacts required by DMD to reconstruct to ∼3.5 Å of the native structure, our method required just 20%–30% contacts to achieve a similar accuracy. Further, the reconstructions with our contact definitions (Ca 9.0 Å, Cb 8.0 Å) for a 30% subset yielded RMSD of 3.25 Å; whereas using Cb 7.5 Å, we get a RMSD of 5.03 Å showing the improved performance of our contact definition.

In case of FT-COMAR, 25% of native distance restraints were required for reconstructing up to ∼4 Å of the native structure [1]. However, a large Ca distance threshold (>15 Å) was used to define the contacts. In comparison, we used a four-fold sparser contact map (20% contacts) and achieved better accuracy (∼3.4 Å). Thus, in the trade-off between the reconstruction accuracy and the size of the subset required for achieving a given accuracy, we observe that our reconstruction method along with our contact definition outperform FT-COMAR and DMD.

### A rational selection of the structural essence

An algorithm capable of picking the structurally important contacts should be able to generate sets with significantly better reconstruction accuracy than by random selection. On the same token, such an algorithm should also require fewer distance restraints as input. To measure the improvement we define a relative performance index (PI) as
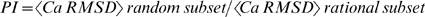
(1)where the size of the random subset equals the rational subset. An algorithm capable of picking a minimal subset that reconstructs better than a random subset scores a PI>1.

The sequence based information in combination with graph-based properties can be used as parameters in devising a rational strategy that identifies the structural essence.

### Long sequence-range distils the structural essence better than short-range

**Figure 3:**
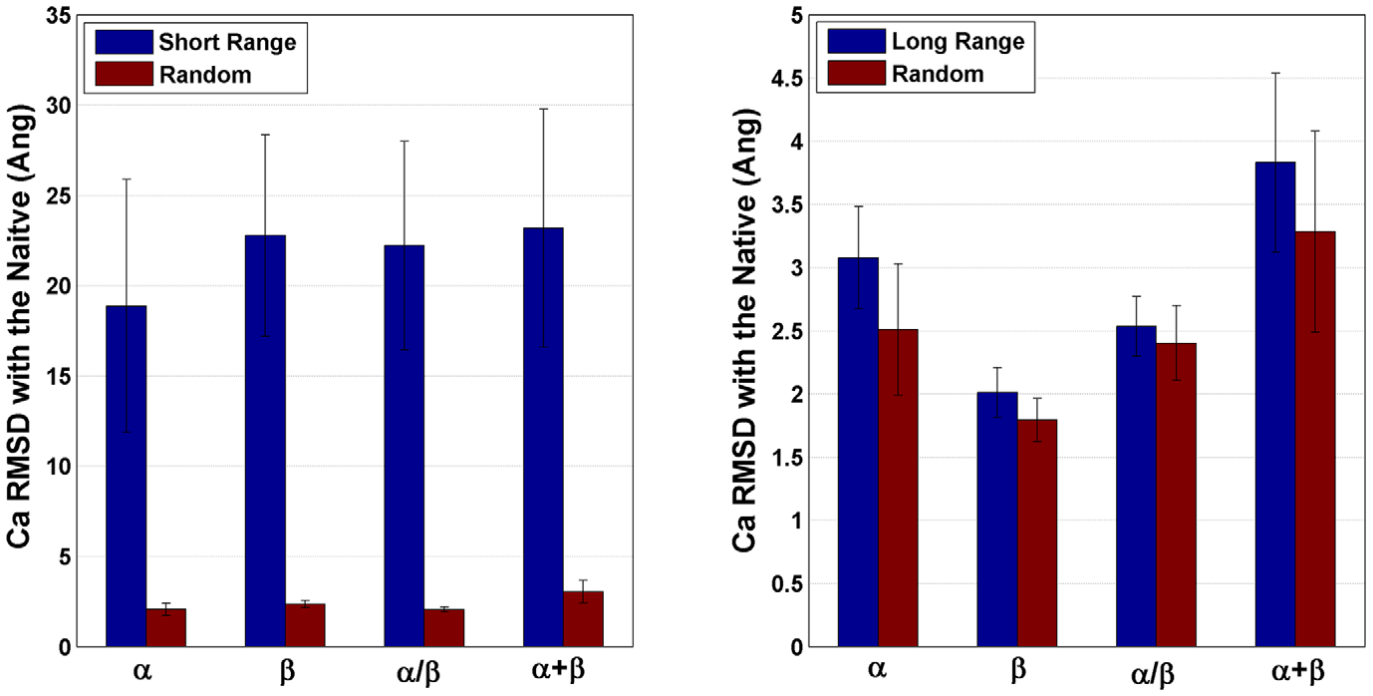
Sequence-range based subset selection. The reconstruction accuracy of the short-range (left) and the long-range subsets (right) are shown (blue). The entire short (SR) and long-range (LR) contacts subsets are used in reconstruction. The comparison is against a random subset of similar size (red). The class average is the average Ca RMSDs from the ensembles (1/4^th^ best models) of every protein. The sizes of the SR and the LR subsets vary slightly in each SCOP class; however the trend was the preserved for both the Ca and the Cb graphs. (The average sizes of Ca graphs:- All α: SR = 62.2%, LR = 37.8%; All β: SR = 51.1%, LR = 49.9%; α/β: SR = 55.5%, LR = 44.5%; α+β: SR = 51.1%, LR = 48.9%).

The sequence-range of a contact is defined as the separation in sequence between the amino acids i and j which are in contact (for details see methods). While contacts from the lower sequence-range are determinants of the secondary structure, the long-range contacts determine the intricacies of the fold and the packing of the tertiary structure. Further, the number of long-range contacts and the long-range contact order influence the folding rate of proteins [18]–[21]. To evaluate the significance of contacts from different sequence-ranges, we selected predefined short and long sequence-range contacts (see methods). The reconstruction accuracies of the chosen subsets compared to similar sized random subset are shown in Figure 3. While the short-range subsets failed to produce a model anywhere near the native structure, the long sequence-range subsets reconstructed significantly better. However, in comparison to the random subsets, these results are not significant and the long-range contacts alone did not achieve a PI>1. Although there is an effect of long-range contacts being more important, a set of long-range contacts alone is not sufficient to capture all the structural information.

### Common neighbourhoods of contacts

**Figure 4:**
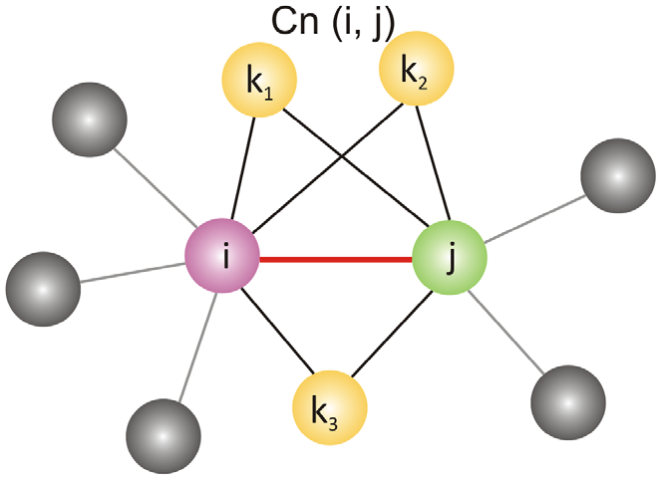
Common Neighbourhood of an edge (Cn(E_ij_)). A contact E_ij_ (red) between nodes i (pink) and j (green) is shown. Let (N_i_) be the neighbours of the i and (N_j_) be neighbours of the j (grey). The CNb of edge (E_ij_) is defined as 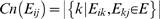
The nodes k_1_, k_2_ and k_3_ (yellow) share edges with nodes i and j. The triangles k_1_, k_2_ and k_3_ make with E_ij_ constitute the CNb triangles of E_ij_.

The concept of common neighbourhoods is used to analyze the significance of an edge and its local neighbourhood to the overall structure and stability of the network. For instance, common neighbourhoods are used in determining packing effects of atoms in crystals [22],[23]. The common neighbourhood (CNb) of a contact is defined in methods and the concept illustrated in Figure 4. For an edge E_ij_ (red) between nodes i (pink) and j (green), the edges formed by i and j with nodes k_1_, k_2_ and k_3_ (yellow) constitute the neighbourhood of E_ij._ The triangle formed by E_ij_ and its neighbours (E_ik_ and E_kj_ (black)) forms the CNb triangle. A contact (red) embedded in its CNb is viewed as a representative of its neighbourhoods (black).

A contact map typically contains many contacts that have few common neighbours and few with many common neighbours. Thus, it is possible to rank contacts based on their CNb sizes. We hypothesize here that contacts that possess more common neighbours are structurally more significant compared to the small neighbourhood counterparts. By stripping the neighbourhoods from the contacts, the ability of contacts to represent their neighbourhood efficiently is tested.

### Can rank based selection outperform a random selection of contacts?

A simple rank-ordered selection of contacts was the initial strategy we employed in selecting structurally important contacts. Native contacts were ranked in the ascending order according to the sequence-range, CNb sizes and increasing fractions (10% to 90%) were selected and reconstructed. The PIs of the rank-ordered subsets are given in Table 2 (the reconstruction accuracies are shown in Figure S1). Even with structurally important parameters like the sequence-range and the CNb sizes, a direct rank-ordered selection failed to distinguish the structurally essential from the non-essential contacts of the protein structure. This is evident when the rank-ordered subsets are visualized in a contact map. The rank-ordering samples only discrete regions of the contact map, while a random selection samples uniformly from different regions of the contact map ensuring better reconstruction. For instance, in the case of the sequence-range ordering, contacts are selected diagonally and clearly carry insufficient information about the protein's tertiary structure (Figure S2). This provides a possible explanation of why contact order ranked selection did not yield a better reconstructing subset for Chen and co-workers [16]. Thus, it is clear that even with a choice of parameters like sequence-range and CNbs that carry significant structural information, the method employed in selecting the best reconstructing subset can be considered as the biggest bottle-neck. Such a method should show better performance when the two parameters are combined in a most efficient way.

**Table 2:** PIs* of common neighbourhood (CNb) and sequence-range rank ordered subsets.

PDB Id	CNb Ranked Subsets					Sequence-range Ranked Subsets				
	**10%**	**30%**	**50%**	**70%**	**90%**	**10%**	**30%**	**50%**	**70%**	**90%**
**1bkr**	0.52	0.67	0.69	0.81	0.94	0.46	0.79	1.07	1.01	1.01
**1odd**	0.37	0.39	0.47	0.75	0.92	0.41	0.70	0.92	1.05	1.08
**1cem**	0.31	0.63	0.71	0.73	0.88	0.28	0.66	0.81	1.05	1.05
**1pzc**	0.41	0.55	0.64	0.75	0.90	0.32	0.50	0.62	0.75	0.67
**1onl**	0.77	0.56	0.60	0.68	0.94	0.38	0.55	0.92	1.19	1.07
**1eur**	0.64	0.53	0.72	0.77	0.89	0.48	0.86	1.07	1.31	1.18
**1e6k**	0.54	0.36	0.54	0.62	0.87	0.54	0.72	1.03	1.15	1.01
**1o8w**	0.31	0.58	0.71	0.80	0.92	0.24	0.47	0.85	1.05	0.94
**1ede**	0.79	1.04	0.92	0.95	1.09	0.65	1.21	1.19	1.34	1.16
**1r9h**	0.37	0.38	0.78	0.88	0.94	0.31	0.53	0.91	1.07	0.98
**1ugm**	0.94	0.77	0.75	0.82	0.92	0.55	0.65	0.87	1.17	0.92
**1iu4**	0.69	0.57	0.69	0.53	0.98	0.28	0.53	0.79	1.05	1.07

*: PIs are calculated for every fraction (10% to 90%) of rank-ordered contacts using Eq. (1).

### Cone-peeling: a rational contact selection algorithm

The CNb sizes of contacts and the sequence-range are effectively combined with other network descriptors like degree in formulating the cone-peeling algorithm. The cone-peeling algorithm is based on the concept of common neighbourhood of edges. The CNb of an edge is defined in Eq. 3 and the concept explained in Figure 4. For any given edge E_ij_, a CNb triangle can be defined with edges E_ik1_ and E_k1j_. Here, we hypothesize that in every neighbourhood triangle if the edges E_ik1_ and E_k1j_ are redundant then every triangle can be reduced to just the edge E_ij_ on some conditions. For instance, if E_ik1_ or E_k1j_ are low sequence-range edges, then E_ij_ can successfully represent E_ik1_ and E_k1j_ and a single edge successfully represents the triangle. Thus, E_ij_ is called the representative edge in its CNb triangle. This is meaningful when visualized in the context of the three-dimensional structure of proteins. Assuming E_ij_ is present in a regular secondary structure such as an alpha-helix, the low sequence-range edges E_ik1_ or E_k1j_ would also be part of the same helix. Thus the presence of the representative edge E_ij_ is sufficient and the edges E_ik1_ and E_k1j_ can be safely deleted.

**Figure 5:**
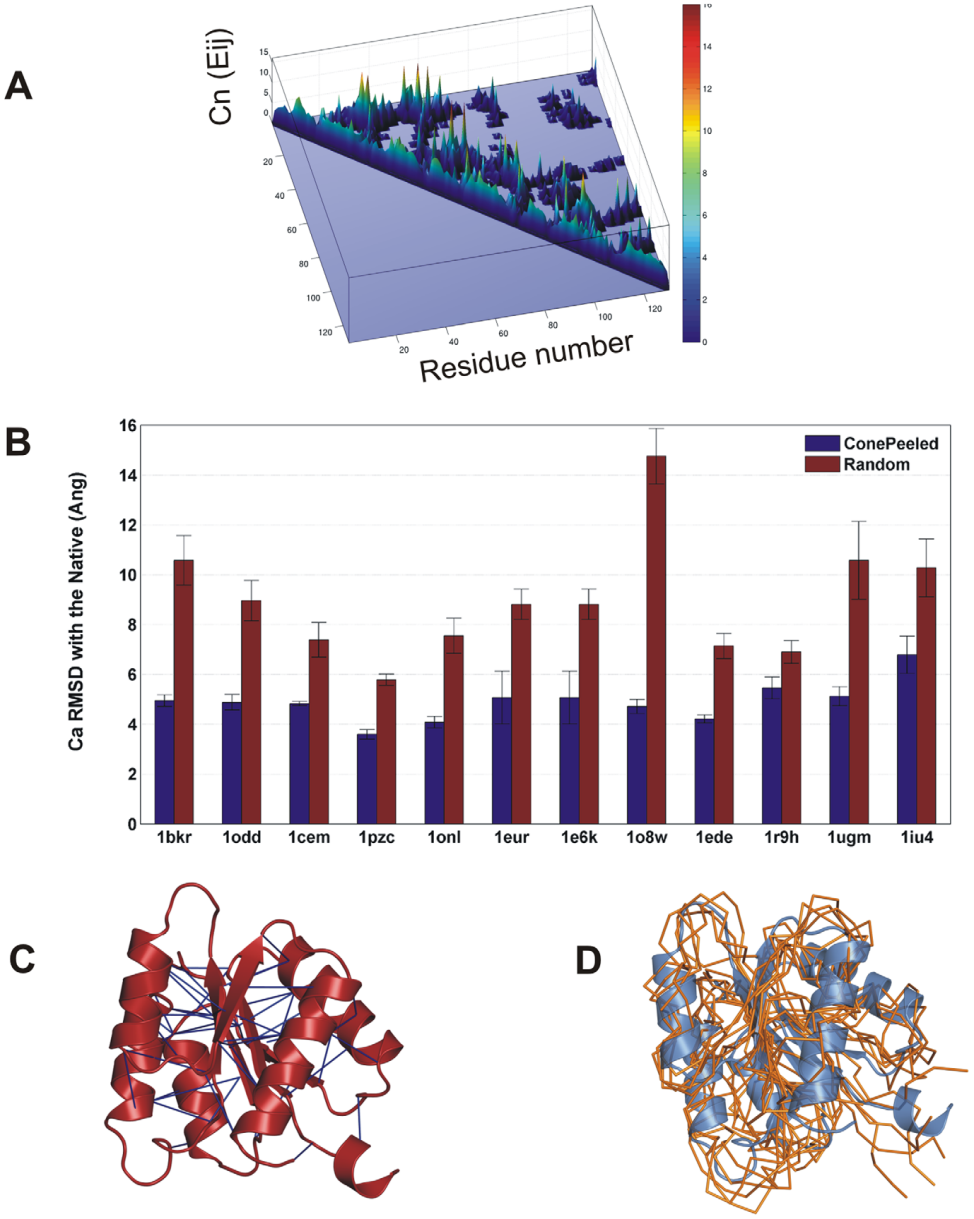
Deriving the structural essence from cone-peeling strategy. A The contact map visualization of the common neighbourhoods. The cone shaped landscape of the CNbs is resultant of low CNb edges occupying the base of the cone, while the high CNb edges occupying the summits. The colour-bar shows the range of the CNb sizes. B The cone-peeling strategy characterizes the structural essence better than random selection. The algorithm selects a subset of native contacts that have high CNb and are also in the long sequence-range and removes all the local contacts. It can be seen that in all the proteins, the subsets selected from cone-peeling (blue) reconstruct better than a similar sized random subset (red) achieving a PI>1 consistently in all the cases. For every protein, the ensemble average Ca RMSD is reported. The sizes of the final subsets and the PIs of the individual proteins are given in Table 1. C The essential contacts (blue) obtained from cone-peeling are highlighted in the native structure of 1e6k (red) using Pymol [29]. With 4.3% of Ca-Ca and 9% of Cb-Cb contacts, the subsets achieve a PI of 1.74. D The overlay of the best reconstructed models onto native structure (1e6k). The models reconstructed from the essential subsets obtained from the cone-peeling algorithm are superposed to the native structure for comparison. The best models selected (in terms of Ca RMSD) are shown in ribbon representation (orange). The native structure is shown in cartoon (blue). The overlaid models show an average Ca RMSD of 4.5 Å to the native structure. In the reconstructed models, only with the essential subsets of contacts, the secondary structural regions are well distinguished from the inter-secondary structural regions.

For the sake of illustration, the CNb triangles in contact maps can be visualized in 3D as occupying the base of a cone while the representative edges occupying the summit (Figure 5A). The height of the cone is defined by the CNb size of the representative edge. In such a scenario, the algorithm peels the cone by deleting local contacts retaining only the summits. This is performed iteratively and every CNbs is replaced with its representative edge in the decreasing order of their degree and the common neighbour sizes of contacts. Thus, the strategy of retaining only higher neighbourhood long-range edges and deleting the low sequence-range neighbours has been implemented in the cone-peeling algorithm. A step-by-step implementation of the cone-peeling algorithm can be obtained from the pseudo code in the methods section.

The long sequence-range and high CNb edges which emerge after cone-peeling is subjected to reconstruction and the accuracies compared with the random subsets in Figure 5B. Our ‘cone-peeled’ subsets from all the SCOP classes exhibit a PI of >1.5 (Table 1). Thus, our ‘cone-peeling’ of local contacts has filtered out the non-essential contacts, while retaining only the essential or structure-determining contacts. It is surprising to note that the minimal subsets of contacts selected from our approach are significantly sparse, on an average comprising about ∼5.8% of Ca and 11.1% of Cb contacts. The cone-peeled subset of contacts for CheY protein (1e6k) is highlighted in Figure 5C. It can be seen that the structural essence as characterized by our algorithm has picked mostly the inter-secondary structural contacts and the contacts from loop regions that are crucial for packing in the protein core, while the ignoring intra-secondary structural contacts and the contacts on the surface. The overlay of five best reconstructed models of CheY (1e6k) onto the native structure is shown in Figure 5D. It can be seen that the secondary structures and the inter-secondary structural regions are distinguished even with a sparse set of Ca and Cb contacts.

With as little as 8% of native contacts (Ca-Ca and Cb-Cb), our algorithm along with our reconstruction pipeline determines the structure of a protein at 4.8 Å (Ca RMSD). At the same time, from a random selection of contacts, roughly twice the number of contacts is necessary to achieve such reconstruction accuracy. Thus, for the first time we report a method that successfully selects native contacts that determine the structure better than a random selection.

## Discussion

We have shown that a structural essence exists among the network of non-covalent contacts of a protein structure. For every protein in our dataset, a subset of 20%–30% native contacts picked at random successfully specifies the global structural features. Further, even by random selection we achieved improvements in the reconstruction accuracy as well as in the size of the subsets over existing methods. This is attributed to the combination of the contact definitions and the reconstruction potential of our method. Thus, up to 70%–80% of the native contact map can be considered dispensable for reconstruction with our method. Hence, the essence affirms the presence of redundant information content in the native contact maps. Redundancy in native contact maps could be envisaged as buffers that would neutralize perturbation effects, which might otherwise destroy the protein fold. This redundancy can be generally compared to the structural effects of mutations in proteins where a comparable trend of greater tolerance to the mutational load exists [24]–[26]. In a broader perspective, this could also be visualized in analogy to genetic knockdown experiments which in many instances does not mediate drastic consequences.

By demonstrating the existence of a rational strategy that outperforms a random contact selection, we disprove of the previous notions, that a random contact selection is sufficient for characterizing the structural essence of a protein. Specifically, we have formulated a rational strategy (cone-peeling) that combines sequence properties and network descriptors to identify essential contacts better than a random subset. Further, we did not discriminate between the contacts based on their secondary or the tertiary structural content. Instead, the structural importance of the selected subset emerged naturally from the choice of the contact properties and the network descriptors and the way in which we have combined the parameters into the algorithm. Even the same parameters used in slightly different ways by other groups did not yield the desired results, emphasizing the success of our algorithm.

The fact that a structural essence of contacts can be described from the native contact network raises further questions about the uniqueness of the essence and its biophysical and the biochemical significance. Do all the essential contacts carry important biological significance? Would mutations to the structural essence have more severe effects? By addressing these issues in future, one can understand of the significance of the essence in the context of protein stability and function.

The filtering of essential contacts from the non-essential ones can be considered as a first step in contact prediction and constraint selection studies. The knowledge gained from the present study that a fraction of native Ca and Cb contacts (8% to 10%) from long sequence-range and high common neighbourhood are sufficient for reconstruction could serve as preliminary guidelines in selecting distance constraints for experimental structure determination problems. However, in its present form, prediction of such contacts from sequence is not easy as the algorithm works by characterizing an essential subset of proteins from the structure. In future, when the essential contacts are analyzed from a large non-redundant set of proteins, the biophysical and structural information obtained from these contacts could be employed as features in machine learning methods to predict essential contacts directly from the sequence. The present paper thus focuses only on the first step in characterizing the essential subsets of contacts from contact maps and further studies are necessary to address the feature selection and the contact prediction issues.

### Conclusions

We have identified a structural essence from the non-covalent contacts of protein, which successfully determines structural features. The essence could be identified as a 20%–30% fraction of native contacts by random selection. We have proposed a rational strategy (cone-peeling) that outperforms a random contact selection and it successfully distilled the structural essence of a protein from the bulk of non-covalent contacts. The cone-peeling combines the sequence and network descriptors to select the essential contacts. The structural essence is only 8% of the native contacts that reconstructs to 4.8 Å (Ca RMSD) to the native structure. However, to attain a similar reconstruction accuracy with random selection about twice the number of contacts is required. Thus, our cone-peeling algorithm is the first rational strategy that characterizes the structural essence in protein structures. The concept of essential contacts in proteins can find further applications in the design of empirical contact potentials, in experimental and theoretical protein structure determination and also in constraint-based comparative sequence design.

## Methods

### Dataset

A non-redundant dataset of proteins is selected from SCOP release 1.73 [27]. Only monomeric, monodomain proteins from the four main SCOP classes and from high populated folds are chosen such that all possible interactions that stabilize the native fold are taken into account. All proteins have resolutions better than 3.0 Å, R-factor lower than 0.3 as well as no missing or ambiguous conformational data (Filippis, personal communication). A subset of 12 proteins, three per SCOP class, is selected from the dataset such that two fall in the size range of 100–120 amino acids and the third is thrice bigger. The PDB codes of the selected proteins are given in Table 1.

### Contact Maps and selection of constraints

The protein structures are represented as graphs with amino acids as nodes and the interactions between the amino acids as edges. Specifically, the contacts between the Ca or Cb atoms are considered as edges. The distance thresholds of 9.0 Å and 8.0 Å are used respectively to define contacts between Ca and Cb atoms (Ca for Gly). The contacts are visualized in a contact map with CMView [CMView: Interactive Contact Map Visualization and Analysis. http://www.molgen.mpg.de/~lappe/cmview/]. All covalent contacts are ignored.

### Model building from distance restraints

The Ca and the Cb contacts are passed as restraints to the distance geometry program (distgeom) of the Tinker molecular dynamics package [28]. The distgeom uses a variation of the EMBED algorithm [15] to find three-dimensional coordinates in agreement with a sparse set of distance restraints. It proceeds by calculating bounds for all pairs of atoms (bounds smoothing), choosing particular distance values from within the bounds (metrization) and then embedding the resulting metric matrix. A final regularization step is performed by which the coordinates obtained are transformed so that their geometry, with respect to bond lengths and angles, is improved. For this purpose, we used the simulated annealing protocol offered by the distgeom program that minimizes an error function that measures the violations to the restraints.

An ensemble of 50 models is generated for every protein in the dataset. Even after enforcing individual amino acids to the L-enantiomer in the refinement from simulated annealing, a solution to the given contact map can still be found such that the fold is ‘mirrored’. These solutions are termed as ‘topological mirrors’ where the global fold possesses the wrong chirality in spite of individual amino acids being in the L-form. The conformations obtained from the distance geometry protocol cannot distinguish such topological mirrors and we overcome this problem by comparing the models with their native structure through Ca RMSD. The Ca RMSD values for the conformation ensemble are found to be distributed bimodally, by simply choosing the lowest fourth of models as ranked by RMSD we are sure to be selecting the correct models (Figure S3). The ensemble average is obtained from the fourth of models with the lowest Ca RMSD.

### Comparison of 3D Reconstruction between Tinker and DMD

We have considered the dataset of 5 proteins (1dd3, 1nxb, 1igd, 1bxy, 1d0d) from Chen and co-workers [12]. For every protein, contacts maps (Ca 9.0 Å, Cb 8.0 Å) were generated and reconstructed with Tinker. However, contact maps were also generated with Cb 7.5 Å, to compare the differences in reconstruction between Tinker and DMD. The covalent contacts are ignored and the ensemble average Ca RMSD is obtained as mentioned earlier.

### Features employed in the selection of contacts

The properties of the contacts employed in selecting a minimal subset of a given contact map are discussed below.

#### Sequence-range

The sequence-range of an edge E_ij_, (S_(E_ij_)), is defined as the separation in sequence between the amino acids i and j which are in contact. 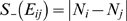
(2)


N_i_, N_j_ are the residue numbers of amino acids i and j. The short and the long-ranges are defined as S_(E_ij_) ≤9 and S_(E_ij_) ≥10 respectively. Subsets were selected with the defined sequence-ranges and reconstructed.

#### Common neighbourhoods

The CNb of an edge (E_ij_) is defined as the set of nearest neighbour edges (ik_1_, jk_1_, ik_2_, jk_2_, ik_3_, jk_3_) common to the amino acids i and j in contact,
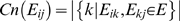
(3)


The CNb of an edge is illustrated in Figure 4. The number of the common neighbourhood triangles of an edge constitutes the CNb size of the edge.

### Cone-peeling algorithm: pseudo code

**Figure 6:**
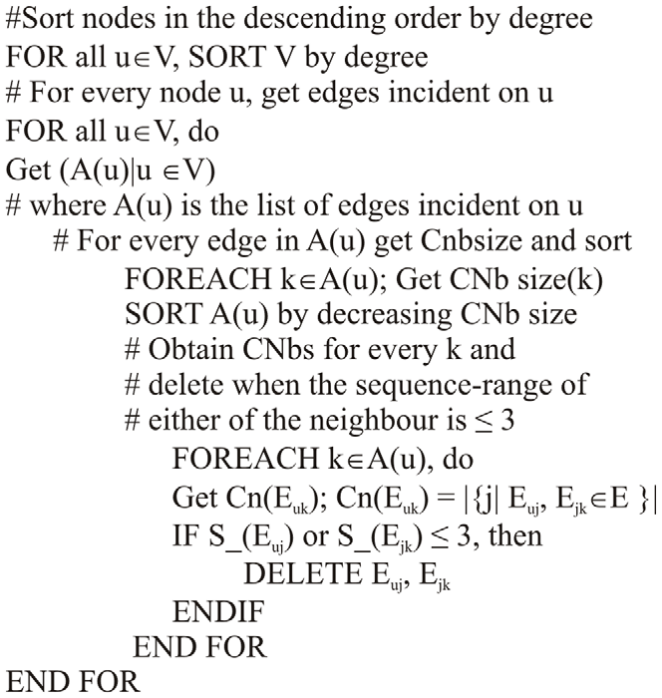
The cone-peeling algorithm.

The algorithm employed in selecting subsets based on peeling of CNbs of contacts is shown in Figure 6.

## Supporting Information

Figure S1 Rank-ordered selection of contacts Increasing fractions (10%–90%) of native contacts are selected by a rank-ordering contacts based on the sequence-range (circle), common neighbourhood (square) properties. In every instance, a similar sized random subset (*) is used to compare the reconstruction accuracies.
(0.27 MB DOC)

Figure S2 Sequence-range based contact selection The contacts selected in a given sequence range is selected across a diagonal in the contact map. Shown are the contacts selected for the sequence-ranges 5 (lower diagonal) and 25 (upper diagonal). The rank-ordered selection based on sequence-range samples contacts along the diagonals and is insufficient for determining the three-dimensional structure.
(0.07 MB DOC)

Figure S3 The distribution of the Ca RMSD for all the models is shown for the cone-peeled subsets. The correct folds were distinguished from the mirrors mainly by filtering using Ca RMSD as it mostly followed a bi modal distribution. For every protein, the lowest fourth of models as ranked by RMSD were selected and the ensemble average was obtained.
(0.25 MB DOC)

Figure S4 GDT-TS Scores of the Cone-Peeled Subsets - GDT-TS Scores of the cone-peeled subsets are shown (blue). The scores of the corresponding random subsets are shown in red
(0.27 MB DOC)

## References

[1] Vassura M, Margara L, Di Lena P, Medri F, Fariselli P (2008). FT-COMAR: fault tolerant three-dimensional structure reconstruction from protein contact maps.. Bioinformatics.

[2] Vendruscolo M, Kussell E, Domany E (1997). Recovery of protein structure from contact maps.. Fold Des.

[3] Mouradov D, Craven A, Forwood JK, Flanagan JU, Garcia-Castellanos R (2006). Modelling the structure of latexin-carboxypeptidase A complex based on chemical cross-linking and molecular docking.. Protein Eng Des Sel.

[4] Petrotchenko EV, Xiao K, Cable J, Chen Y, Dokholyan NV (2008). BiPS, a photo-cleavable, isotopically-coded, fluorescent crosslinker for structural proteomics.. Mol Cell Proteomics.

[5] Alexander N, Bortolus M, Al-Mestarihi A, McHaourab H, Meiler J (2008). De novo high-resolution protein structure determination from sparse spin-labeling EPR data.. Structure.

[6] Young MM, Tang N, Hempel JC, Oshiro CM, Taylor EW (2000). High throughput protein fold identification by using experimental constraints derived from intramolecular cross-links and mass spectrometry.. Proc Natl Acad Sci U S A.

[7] Aszodi A, Gradwell MJ, Taylor WR (1995). Global fold determination from a small number of distance restraints.. J Mol Biol.

[8] DePristo MA, De Bakker PI, Shetty RP, Blundell TL (2003). Discrete restraint-based protein modeling and the Calpha-trace problem.. Protein Sci.

[9] Furnham N, de Bakker PI, Gore S, Burke DF, Blundell TL (2008). Comparative modelling by restraint-based conformational sampling.. BMC Struct Biol.

[10] Lin M, Lu HM, Chen R, Liang J (2008). Generating properly weighted ensemble of conformations of proteins from sparse or indirect distance constraints.. J Chem Phys.

[11] Skolnick J, Kolinski A, Ortiz AR (1997). MONSSTER: a method for folding globular proteins with a small number of distance restraints.. J Mol Biol.

[12] Smith-Brown MJ, Kominos D, Levy RM (1993). Global folding of proteins using a limited number of distance constraints.. Protein Eng.

[13] Wolff K, Vendruscolo M, Porto M (2008). A stochastic method for the reconstruction of protein structures from one-dimensional structural profiles.. Gene.

[14] Vassura M, Margara L, Di Lena P, Medri F, Fariselli P (2008). Reconstruction of 3D structures from protein contact maps.. IEEE/ACM Trans Comput Biol Bioinform.

[15] Crippen GH, Havel TF (1988). Distance Geometry and Molecular Conformation..

[16] Chen Y, Ding F, Dokholyan NV (2007). Fidelity of the protein structure reconstruction from inter-residue proximity constraints.. J Phys Chem B.

[17] Porto M, Bastolla U, Roman HE, Vendruscolo M (2004). Reconstruction of protein structures from a vectorial representation.. Phys Rev Lett.

[18] Bagler G, Sinha S (2007). Assortative mixing in Protein Contact Networks and protein folding kinetics.. Bioinformatics.

[19] Gromiha MM, Selvaraj S (2004). Inter-residue interactions in protein folding and stability.. Prog Biophys Mol Biol.

[20] Baker D (2000). A surprising simplicity to protein folding.. Nature.

[21] Bonneau R, Ruczinski I, Tsai J, Baker D (2002). Contact order and ab initio protein structure prediction.. Protein Sci.

[22] Luemmen N, Kraska T (2007). Common Neighbour Analysis for binary systems.. Modelling and Simulation in Material Science and Engineering.

[23] Tsusuki H, Branicio PS, Rino JP (2007). Structural characterization of deformed crystals by analysis of common atomic neighborhood. Computer Physics Communications..

[24] Besenmatter W, Kast P, Hilvert D (2007). Relative tolerance of mesostable and thermostable protein homologs to extensive mutation.. Proteins.

[25] Woycechowsky KJ, Choutko A, Vamvaca K, Hilvert D (2008). Relative tolerance of an enzymatic molten globule and its thermostable counterpart to point mutation.. Biochemistry.

[26] Bloom JD, Lu Z, Chen D, Raval A, Venturelli OS (2007). Evolution favors protein mutational robustness in sufficiently large populations.. BMC Biol.

[27] Murzin AG, Brenner SE, Hubbard T, Chothia C (1995). SCOP: a structural classification of proteins database for the investigation of sequences and structures.. J Mol Biol.

[28] Ponder JW (2004). Software Tools for Molecular Design, User's Guide for Version 4.2..

[29] Delano WL (2002). The PyMOL Molecular Graphics System..

