# Supplementary material for: Defining an Essence of Structure Determining Residue Contacts in Proteins
Source: PLoS Comput Biol. 2009 Dec 4;5(12):e1000584. doi: 10.1371/journal.pcbi.1000584 (PMC2778133; doi:10.1371/journal.pcbi.1000584)
Supplement: Figure S1 — Rank-ordered selection of contacts Increasing fractions (10%–90%) of native contacts are selected by a rank-ordering contacts based on the sequence-range (circle), common neighbourhood (square) properties. In every instance, a similar sized random subset (*) is used to compare the reconstruction accuracies. (0.27 MB DOC) [file pcbi.1000584.s001.doc]

**Figure S1**


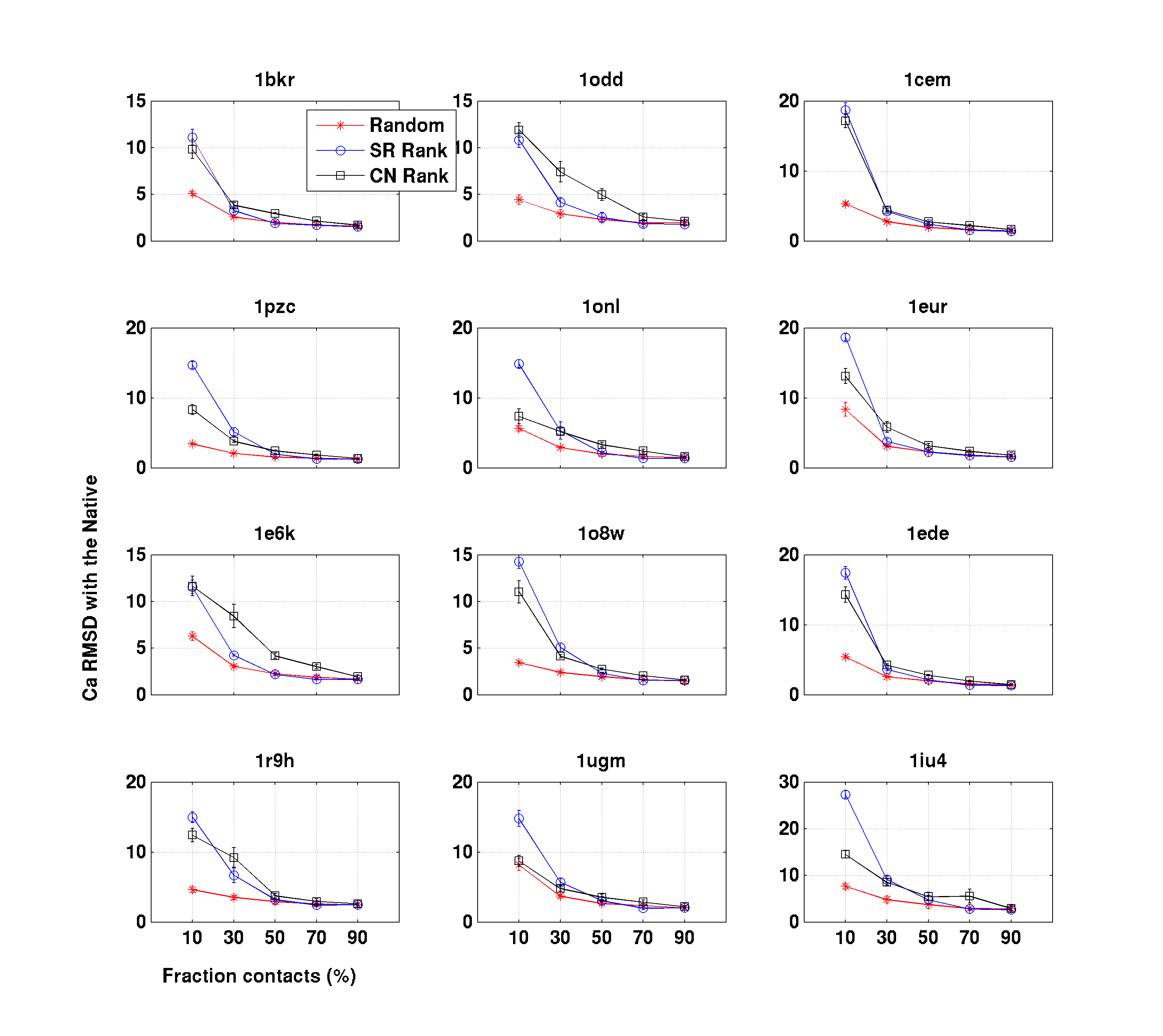


**Rank-ordered selection of contacts**: Increasing fractions (10% - 90%) of native contacts are selected by rank-ordering based on the sequence-range (‘circle’) and common neighbourhood (‘square’) properties. In every instance, a similar sized random subset (‘*’) is used to compare the reconstruction accuracies.
