# Supplementary material for: Defining an Essence of Structure Determining Residue Contacts in Proteins
Source: PLoS Comput Biol. 2009 Dec 4;5(12):e1000584. doi: 10.1371/journal.pcbi.1000584 (PMC2778133; doi:10.1371/journal.pcbi.1000584)
Supplement: Figure S2 — Sequence-range based contact selection The contacts selected in a given sequence range is selected across a diagonal in the contact map. Shown are the contacts selected for the sequence-ranges 5 (lower diagonal) and 25 (upper diagonal). The rank-ordered selection based on sequence-range samples contacts along the diagonals and is insufficient for determining the three-dimensional structure. (0.07 MB DOC) [file pcbi.1000584.s002.doc]

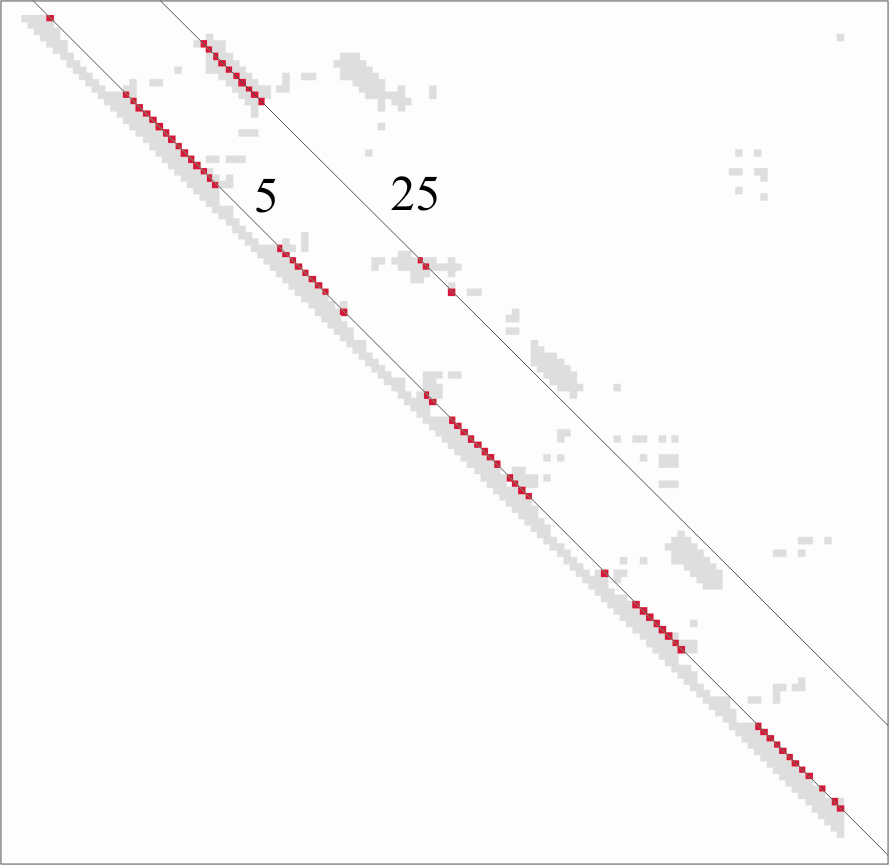
 **Figure S2**

**Sequence-range based contact selection**: The contacts selected in a given sequence range (red) is selected across a diagonal in the contact map. Shown are the contacts selected for the sequence-ranges 5 (lower diagonal) and 25 (upper diagonal). The rank-ordered selection based on sequence-range samples contacts only along the diagonals and is insufficient for determining the three-dimensional structure.
