# Supplementary material for: Defining an Essence of Structure Determining Residue Contacts in Proteins
Source: PLoS Comput Biol. 2009 Dec 4;5(12):e1000584. doi: 10.1371/journal.pcbi.1000584 (PMC2778133; doi:10.1371/journal.pcbi.1000584)
Supplement: Figure S3 — The distribution of the Ca RMSD for all the models is shown for the cone-peeled subsets. The correct folds were distinguished from the mirrors mainly by filtering using Ca RMSD as it mostly followed a bi modal distribution. For every protein, the lowest fourth of models as ranked by RMSD were selected and the ensemble average was obtained. (0.25 MB DOC) [file pcbi.1000584.s003.doc]

**Figure S3**

**
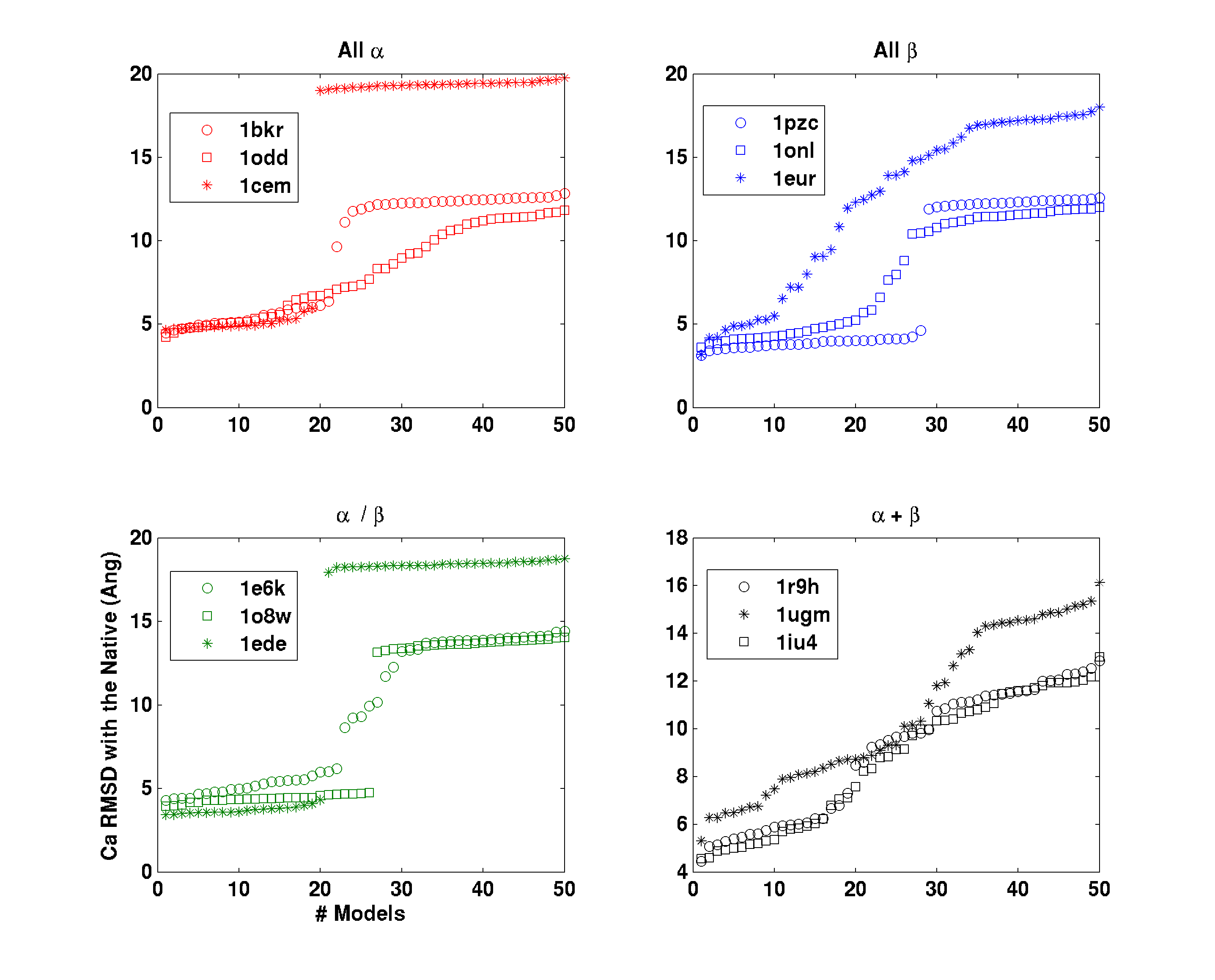
**

The distribution of the Ca RMSD for all the models is shown for the cone-peeled subsets. The correct folds were distinguished from the mirrors mainly by filtering using Ca RMSD as it mostly followed a bi modal distribution. For every protein, the lowest fourth of models as ranked by RMSD were selected and the ensemble average was obtained.
