# Supplementary material for: Defining an Essence of Structure Determining Residue Contacts in Proteins
Source: PLoS Comput Biol. 2009 Dec 4;5(12):e1000584. doi: 10.1371/journal.pcbi.1000584 (PMC2778133; doi:10.1371/journal.pcbi.1000584)
Supplement: Figure S4 — GDT-TS Scores of the Cone-Peeled Subsets - GDT-TS Scores of the cone-peeled subsets are shown (blue). The scores of the corresponding random subsets are shown in red (0.27 MB DOC) [file pcbi.1000584.s004.doc]

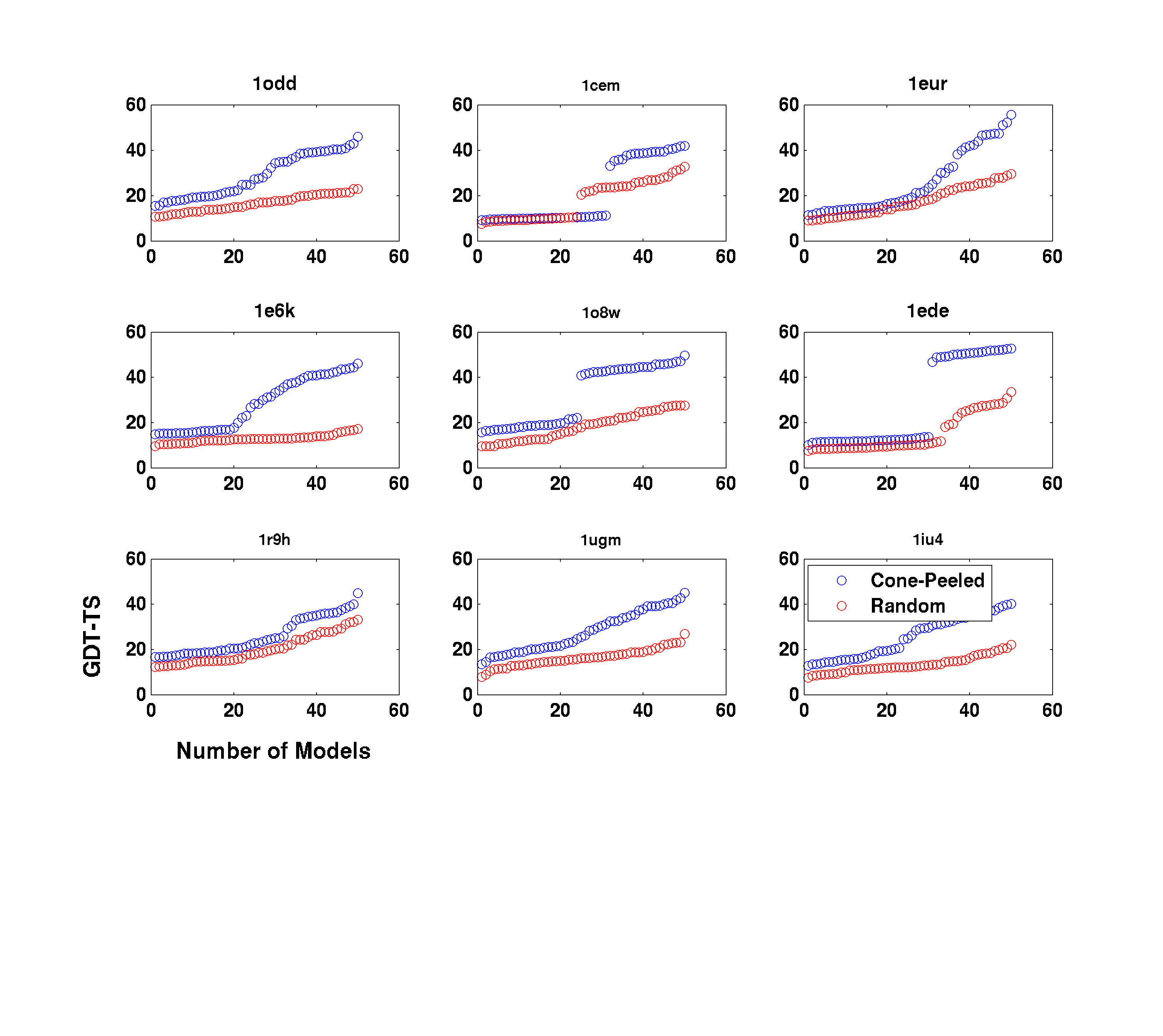


**Figure S4**

**Distribution of the GDT-TS of the Cone-Peeled Subsets**: The GDT-TS of the cone-peeled subsets are shown (blue). The scores of the corresponding random subsets are shown in red. The distribution is shown for all the models generated for a protein.
